# The Ultrashort Mental Health Screening Tool Is a Valid and Reliable Measure With Added Value to Support Decision-making

**DOI:** 10.1097/CORR.0000000000002718

**Published:** 2023-06-20

**Authors:** Robbert M. Wouters, Willemijn A. de Ridder, Harm P. Slijper, Guus M. Vermeulen, Steven E. R. Hovius, Ruud W. Selles, the Hand-Wrist Study Group, Mark J. W. van der Oest

**Affiliations:** a Members of the Hand-Wrist Study Group are listed in an Appendix at the end of this article.; 1 Department of Plastic, Reconstructive, and Hand Surgery, Erasmus MC, Rotterdam, the Netherlands; 2 Department of Rehabilitation Medicine, Erasmus MC, Rotterdam, the Netherlands; 3 Hand and Wrist Center, Xpert Clinics, Eindhoven, the Netherlands; 4 Center for Hand Therapy, Xpert Handtherapie, Eindhoven, the Netherlands; 5 Department of Plastic, Reconstructive, and Hand Surgery, Radboudumc University Hospital, Nijmegen, the Netherlands

## Abstract

**Background:**

Mental health influences symptoms, outcomes, and decision-making in musculoskeletal healthcare. Implementing measures of mental health in clinical practice can be challenging. An ultrashort screening tool for mental health with a low burden is currently unavailable but could be used as a conversation starter, expectation management tool, or decision support tool.

**Questions/purposes:**

(1) Which items of the Pain Catastrophizing Scale (PCS), Patient Health Questionnaire (PHQ-4), and Brief Illness Perception Questionnaire (B-IPQ) are the most discriminative and yield a high correlation with the total scores of these questionnaires? (2) What is the construct validity and added clinical value (explained variance for pain and hand function) of an ultrashort four-item mental health screening tool? (3) What is the test-retest reliability of the screening tool? (4) What is the response time for the ultrashort screening tool?

**Methods:**

This was a prospective cohort study. Data collection was part of usual care at Xpert Clinics, the Netherlands, but prospective measurements were added to this study. Between September 2017 and January 2022, we included 19,156 patients with hand and wrist conditions. We subdivided these into four samples: a test set to select the screener items (n = 18,034), a validation set to determine whether the selected items were solid (n = 1017), a sample to determine the added clinical value (explained variance for pain and hand function, n = 13,061), and a sample to assess the test-retest reliability (n = 105). Patients were eligible for either sample if they completed all relevant measurements of interest for that particular sample. To create an ultrashort screening tool that is valid, reliable, and has added value, we began by picking the most discriminatory items (that is, the items that were most influential for determining the total score) from the PCS, PHQ-4, and B-IPQ using chi-square automated interaction detection (a machine-learning algorithm). To assess construct validity (how well our screening tool assesses the constructs of interest), we correlated these items with the associated sum score of the full questionnaire in the test and validation sets. We compared the explained variance of linear models for pain and function using the screening tool items or the original sum scores of the PCS, PHQ-4, and B-IPQ to further assess the screening tool’s construct validity and added value. We evaluated test-retest reliability by calculating weighted kappas, ICCs, and the standard error of measurement.

**Results:**

We identified four items and used these in the screening tool. The screening tool items were highly correlated with the PCS (Pearson coefficient = 0.82; p < 0.001), PHQ-4 (0.87; p < 0.001), and B-IPQ (0.85; p < 0.001) sum scores, indicating high construct validity. The full questionnaires explained only slightly more variance in pain and function (10% to 22%) than the screening tool did (9% to 17%), again indicating high construct validity and much added clinical value of the screening tool. Test-retest reliability was high for the PCS (ICC 0.75, weighted kappa 0.75) and B-IPQ (ICC 0.70 to 0.75, standard error of measurement 1.3 to 1.4) items and moderate for the PHQ-4 item (ICC 0.54, weighted kappa 0.54). The median response time was 43 seconds, against more than 4 minutes for the full questionnaires.

**Conclusion:**

Our ultrashort, valid, and reliable screening tool for pain catastrophizing, psychologic distress, and illness perception can be used before clinician consultation and may serve as a conversation starter, an expectation management tool, or a decision support tool. The clinical utility of the screening tool is that it can indicate that further testing is warranted, guide a clinician when considering a consultation with a mental health specialist, or support a clinician in choosing between more invasive and less invasive treatments. Future studies could investigate how the tool can be used optimally and whether using the screening tool affects daily clinic decisions.

**Level of Evidence:**

Level II, diagnostic study.

## Introduction

In musculoskeletal healthcare, patient mental health has gained attention in recent years. Numerous studies have demonstrated that mental health factors influence symptoms, outcomes, and treatment choices [3-5, 8, 9, 12, 14-16, 18, 19, 25, 28, 30, 31, 33, 35, 36]. For example, patients with thumb-base osteoarthritis scheduled for surgery have worse psychologic profiles than their nonsurgical counterparts [37], suggesting that domains of mental health play an important role in choosing between surgical and nonsurgical treatment. Important mental health domains include pain catastrophizing, psychologic distress (anxiety and depression), and illness perceptions. Given the relevance of mental health in many musculoskeletal conditions, it is valuable to routinely examine one’s mental health to support personalized and value-based healthcare and facilitate shared decision-making [1, 2, 24]. Several patient-reported measures of mental health are available, including the Pain Catastrophizing Scale (PCS) [29], the four-item Patient Health Questionnaire (PHQ-4) [22], and the Brief Illness Perception Questionnaire (B-IPQ) [6, 10, 23], adding up to 25 questions if one would obtain a (relatively) complete picture of a patient’s mental health. Implementing these or similar measures in clinical practice can be challenging. Using mental health measures in addition to standard outcome sets (such as for hand and wrist conditions [34]) requires greater time investment from patients and adds to the burden of routine outcome measurements.

Hypothetically, questionnaires with fewer items may yield a higher compliance rate. Another issue of implementing measures of mental health in daily clinical practice is that patients may not understand why they have to complete these questionnaires if, in their opinion, they have very objectifiable symptoms because of a specific physical condition (such as osteoarthritis). Consequently, patients may feel that using these measures to evaluate mental health is inappropriate. Reducing the number of questions while obtaining a valid and reliable picture of a patient’s mental health could be a solution. This would also be helpful for clinicians, because many clinicians in musculoskeletal healthcare have little or no time for an in-depth evaluation of mental health during a consultation, and they may also lack the skills for such conversations.

There is a need for a short screening tool that provides an accurate view of patients’ mental health with a low patient and clinician burden to overcome these issues. Ideally, such a screening tool would be used before a primary clinician consultation to guide the consultation. A screening tool for mental health would have great clinical relevance because it can be used as a conversation starter, expectation management tool, or decision support tool. For example, it could enable clinicians to discuss the patient’s thoughts and feelings and the influence of those thoughts and feelings on perceived symptoms and treatment outcomes, or it may inform the decision to refer a patient to a mental health specialist.

Therefore, we asked: (1) Which items of the Pain Catastrophizing Scale (PCS), Patient Health Questionnaire (PHQ-4), and Brief Illness Perception Questionnaire (B-IPQ) are the most discriminative and yield a high correlation with the total scores of these questionnaires? (2) What is the construct validity and added clinical value (explained variance for pain and hand function) of an ultrashort four-item mental health screening tool? (3) What is the test-retest reliability of the screening tool? (4) What is the response time for the ultrashort screening tool?

## Patients and Methods

### Study Design and Setting

This prospective cohort study followed the STrengthening the Reporting of Observational studies in Epidemiology statement [32]. Data were collected at Xpert Clinics, comprising 25 specialized treatment centers in the Netherlands for hand surgery and therapy. Patient care is reimbursed by Dutch basic insurance. Xpert Clinics currently employs 27 hand surgeons and more than 150 hand therapists. All hand surgeons are certified by the Federation of European Societies for Surgery of the Hand or are fellowship-trained. Data collection was part of usual care, but prospective measurements were added to this study. In the routine outcome measurement system, a measurement track is assigned to each patient, including predefined measurements at predefined timepoints. Details on our routine outcome measurement system are described elsewhere [27].

### Participants

**Fig. 1:**
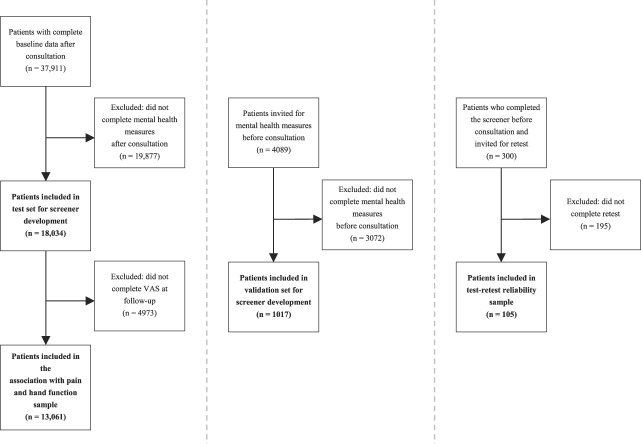
This flowchart of the study shows the inclusion and exclusion criteria.

We used four samples. The first was a test set in which we developed the screening tool and first assessed construct validity (how well our screening tool assesses the constructs of interest). Between September 2017 and January 2022, we treated 37,911 patients for various hand and wrist conditions. Of those, we considered adult patients who completed the mental health measures after clinician consultation as part of their routine outcome measurement as potentially eligible for the test set. These measures were baseline measurements for patients scheduled for either nonsurgical or surgical treatment. Based on that, 48% (18,034) were included in the test set; 52% (19,877) were excluded because of missing data (Fig. 1). The second sample was a validation set and was used to determine whether the selected items were solid (1017). Between September 2017 and January 2022, we invited an additional 4089 patients with various hand and wrist conditions to complete the mental health measures before consultation. We considered all patients who completed these measures eligible for the validation set. Based on that, 25% (1017) were included in the validation set, and the remaining 75% (3071) were excluded because of missing data.

To further assess construct validity and added clinical value (explained variance for pain and hand function), we used a third sample to assess the association of the screening tool items with pain and hand function at baseline and at 3 months of follow-up. We considered all patients from the test set who also completed the VAS for pain and function at baseline and 3 months eligible for this sample. We included 72% (13,061) of the sample regarding the explained variance for pain and hand function and excluded 28% (4973).

We used a fourth sample to assess the test-retest reliability. In January 2022, we invited 300 patients who had completed the mental health screening tool before clinician consultation to complete it again within 5 to 10 days. This had to be before their scheduled hand surgeon consultation. We included 35% (105) of the test-retest reliability sample. The median interval between measures was 6 days (range 5 to 10).

We assessed whether responders and nonresponders in the sample systematically differed regarding the association between the screening tool items and pain and hand function and the test-retest reliability. In the sample of the explained variance for pain and hand function, we defined responders as patients who completed all measures at baseline (sociodemographics and mental health questionnaires) and 3 months of follow-up (the VAS), whereas nonresponders were patients who only completed baseline measures. In the test-retest reliability sample, responders were patients who completed the primary test and retest, whereas nonresponders were patients who only completed the primary test. We calculated the standardized mean difference between responders and nonresponders. We only found small, clinically irrelevant differences in age and assigned treatment track between responders and nonresponders in the sample of the explained variance for pain and hand function (Supplemental Table 1; http://links.lww.com/CORR/B138). We found no differences between responders and nonresponders in the test-retest reliability sample (Supplemental Table 2; http://links.lww.com/CORR/B176).

After applying the eligibility criteria, we included 18,034 patients in the test set, 1017 patients in the validation set, 13,061 patients in the sample regarding the added value of the screening tool (that is, its association with pain and function), and 105 patients in the test-retest reliability sample (Fig. 1). The demographic characteristics of these patients were representative of a general population of patients with hand and wrist conditions (Table 1).

**Table 1.** Baseline characteristics of the study sample

Variable	Sample 1: Test set (n = 18,034)	Sample 2: Validation set (n = 1017)	Sample 3: Association with pain and hand function (n = 13,061)	Sample 4: Test-retest reliability (n = 105)
Age in years	54 ± 15	57 ± 15	55 ± 14	56 ± 16
Sex, female	65 (11,797)	63 (644)	66 (8602)	60 (63)
Treatment track				
Thumb regular	16 (2912)		15 (1977)	
Thumb extended	7 (1191)		7 (964)	
Dupuytren	9 (1561)		9 (1232)	
Wrist regular	22 (3958)		20 (2669)	
Wrist extended	8 (1388)		8 (1103)	
Finger regular	19 (3441)		19 (2518)	
Finger extended	3 (524)		3 (378)	
Nerve compression or decompression	17 (3059)		17 (2220)	
Duration of symptoms in months	19 ± 38	16 ± 30	19 ± 39	23 ± 66
Type of work				
Unemployed (including retired)	34 (6110)	39 (396)	35 (4622)	42 (44)
Light physical labor (such as office work)	29 (5140)	24 (244)	28 (3699)	19 (20)
Moderate physical labor (such as working in a store)	27 (4790)	21 (217)	26 (3371)	29 (30)
Heavy physical labor (such as working in construction)	11 (1994)	16 (160)	11 (1369)	11 (11)
Treated or affected side^a^				33 (35) 37 (39) 30 (31)
Left	41 (7455)	30 (308)	42 (5483)	
Right	54 (9661)	37 (376)	54 (6995)	
Both	5 (918)	33 (333)	5 (583)	
Dominant hand				11 (12) 81 (85) 8 (8)
Left	8 (1492)	10 (103)	8 (1076)	
Right	89 (16,039)	83 (841)	89 (11,601)	
Both	3 (503)	7 (73)	3 (384)	
Second opinion, no	96 (17,230)	85 (862)	95 (12,461)	89 (93)
PHQ-4 total score (scores can range from 0 to 12)	1.4 ± 2.3	1.7 ± 2.6	1.3 ± 2.2	
PCS total score (scores can range from 0 to 52)	11.2 ± 9.7	13.5 ± 10.3	11 ± 9.5	
B-IPQ total score (scores can range from 0 to 80)	37.0 ± 11.5	40.3 ± 11.0	36.8 ± 11.5	

Data are presented as % (n) or mean ± SD.

a For the validation set (Sample 2) and the test-retest reliability sample (Sample 4), the patient is asked which side is affected, whereas the values in Samples 1 and 3 reflect the side that is treated.

### Variables, Data Sources, and Measurement

We measured pain catastrophizing using the 13-item PCS (score range 0 to 52; higher scores indicate more catastrophizing) [29], psychologic distress using the four-item PHQ-4 (score range 0 to 12; higher scores indicate a potential anxiety or depression disorder) [22], and illness perception using the eight-item B-IPQ (total score range 0 to 80; higher scores indicate more negative illness perception) [6, 10, 23]. These are all valid and reliable instruments [6, 10, 22, 23, 29].

Sociodemographic characteristics collected at baseline included age, sex, measurement track (a predefined set of measurements at predefined timepoints based on the patient’s diagnosis) [27], duration of symptoms, type of work, affected side, dominant hand, and whether a second opinion was sought. Lastly, we used the VAS, which is valid and reliable [17], to measure pain (range 0 to 100, higher scores indicate more pain) and hand function (range 0 to 100, lower scores indicate worse hand function) at baseline and 3 months.

### Sample Size

Although large sample sizes (ideally more than 1000) [26] are required for chi-square automated interaction detection, we found no recommendations for sample size. Therefore, we used a convenience sample for the test set (postconsultation) and aimed to include more than 1000 participants. For the test-retest reliability sample, at least 50 participants are recommended [11], which is well below our sample of 105 participants.

### Ethical Approval

Ethical approval for this study was obtained from Erasmus MC, Rotterdam, the Netherlands.

### Statistical Analysis

We used a chi-square automated interaction detection [20] machine-learning algorithm in the test set (Sample 1) to select the items for the screening tool. In each questionnaire, the chi-square automated interaction detection algorithm determined which item has the most discriminative power for the sum score of that questionnaire. These items were subsequently picked, and we calculated the Pearson correlation between these items and the associated sum score to assess the construct validity. To ensure high construct validity of the screening tool, we proposed that there should at least be a very strong correlation (that is, Pearson ≥ 0.80) [13] between the selected items and the sum score of the particular questionnaire in the test set for each construct of interest (such as pain catastrophizing, psychologic distress, or illness perception). We also calculated the Pearson correlation between the selected items and the sum scores in the validation set (Sample 2) to investigate whether the selected items were solid, also aiming for a very strong correlation (that is, Pearson ≥ 0.80) in the validation set for the screening tool to be accurate.

To further assess the construct validity, we built linear regression models using Sample 3 to assess the explained variance of the screening tool items, with VAS pain during physical load and VAS hand function as dependent variables, both of which were measured at baseline and 3 months, adding up to four models. We built four additional models for the same dependent variables but with the total scores of the full questionnaires (the full PCS, PHQ-4, and B-IPQ) and compared the multiple r-squared of these models with those of the models only using the screening tool. In the models using the 3-month measurement as the dependent variable, we adjusted for baseline scores by adding the baseline score first in the model, because these are usually associated with the follow-up score [27]. By doing this, the explained variance we report is more reliably independent from the baseline scores.

For the test-retest reliability, we calculated the weighted kappa and ICCs for categorical items and ICCs and the standard error of measurement for continuous items.

## Results

### Screening Tool Development (Most Discriminative Item Selection and Correlation With Total Scores)

**Fig. 2:**
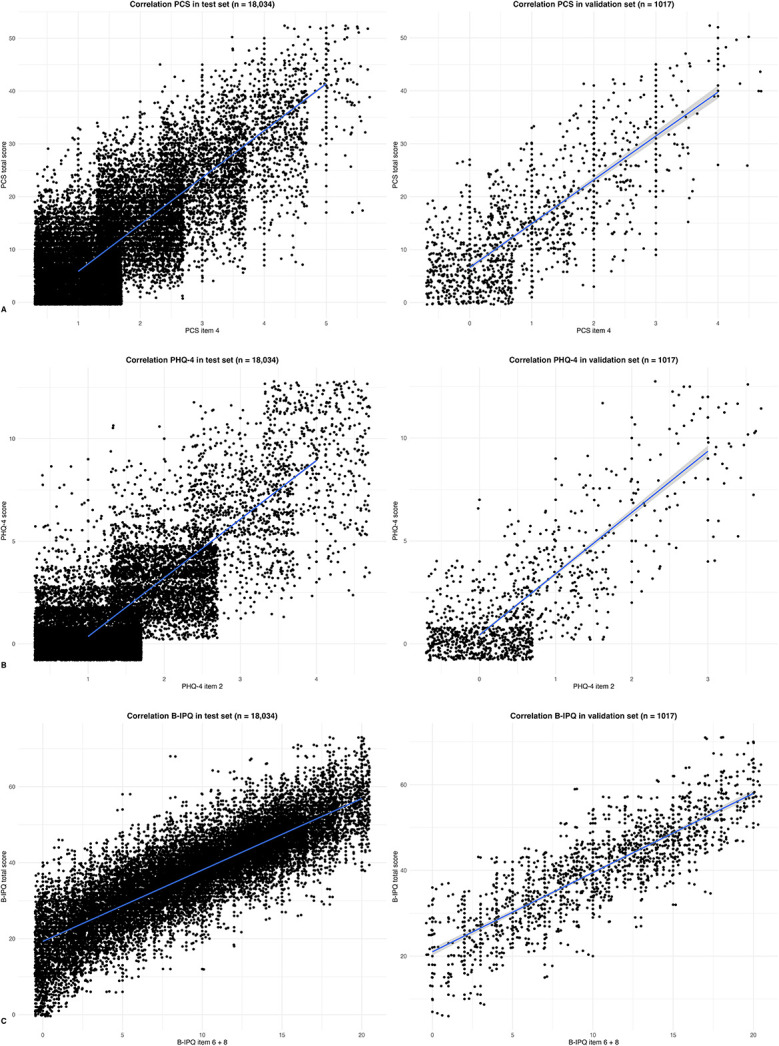
These scatterplots demonstrate the correlation between the screening tool items and the sum scores. (**A**) For item 4 of the PCS and PCS total score, the Pearson correlation was 0.82 (95% CI 0.81 to 0.82; p < 0.001) in the test set (left) and 0.81 (95% CI 0.79 to 0.83; p < 0.001) in the validation set (right). (**B**) For item 2 of the PHQ-4 and PHQ-4 total score, the Pearson correlation was 0.87 (95% CI 0.86 to 0.88; p < 0.001) in the test set (left) and 0.88 (95% CI 0.86 to 0.89; p < 0.001) in the validation set (right). (**C**) For the B-IPQ items 6 and 8 and B-IPQ total score, the Pearson correlation was 0.85 (95% CI 0.85 to 0.86; p < 0.001) in the test set (left) and 0.85 (95% CI 0.83 to 0.86; p < 0.001) in the validation set (right). A color image accompanies the online version of this article.

The chi-square automated interaction detection algorithm selected four items for the final screening tool (Table 2). For pain catastrophizing, the chi-square automated interaction detection algorithm selected item 4 (“When I’m in pain, it’s awful and I feel that it overwhelms me”) of the PCS (test set: Pearson correlation 0.82 [95% CI 0.81 to 0.82]; p < 0.001, validation set: 0.81 [0.79 to 0.83]; p < 0.001) (Fig. 2A). Item 2 of the PHQ-4 (“Not being able to stop or control worrying”) was selected for psychologic distress (test set: 0.87 [95% CI 0.86 to 0.88]; p < 0.001, validation set: 0.88 [95% CI 0.86 to 0.89]; p < 0.001) (Fig. 2B). Two items of the B-IPQ were required to obtain a correlation greater than 0.80, resulting in the selection of items 6 (concern: “How concerned are you about your illness?”) and 8 (emotional response: “How much does your illness affect you emotionally? (such as, does it make you angry, scared, upset or depressed?)”) (test set: 0.85 [95% CI 0.85 to 0.86]; p < 0.001, validation set: 0.85 [95% CI 0.83 to 0.86]; p < 0.001) (Fig. 2C) from the chi-square automated interaction detection algorithm.

**Table 2.** The final screening tool for mental health

Item	Question	Score range	Response options
PCS item 4	When I’m in pain, it’s awful and I feel that it overwhelms me	0-4	Not at all
			To a slight degree
			To a moderate degree
			To a great degree
			All the time
PHQ-4 item 2	Over the last 2 weeks, how often were you not able to stop or control worrying?	0-3	Not at all
			Several days
			More than half the days
			Nearly every day
B-IPQ item 6	How concerned are you about your illness?	0-10	Anchors: “Not at all concerned” (0) to “extremely concerned” (10)
B-IPQ item 8	How much does your illness affect you emotionally (e.g., does it make you angry, scared, upset or depressed)?	0-10	Anchors: “Not at all affected emotionally” (0) to “extremely affected emotionally” (10)

### Construct Validity and Added Clinical Value (Association With Pain and Function)

The screening tool explained 17% of the variance in pain at baseline and 14% at 3 months. For function, this was 10% at baseline and 9% at 3 months. The full questionnaires performed only slightly better and explained 22% of the variance in pain at baseline and 15% at 3 months. For function, this was 13% at baseline and 10% at 3 months. Combined with the abovementioned correlations, this indicates the screening tool has high construct validity and added clinical value.

### Test-retest Reliability

There was a high test-retest reliability for PCS item 4 (ICC 0.75 [95% CI 0.66 to 0.83], weighted kappa 0.75 [95% CI 0.66 to 0.84]) and B-IPQ items 6 (ICC 0.70 [95% CI 0.59 to 0.79]; standard error of measurement 1.4) and 8 (ICC 0.75 [95% CI 0.65 to 0.82]; standard error of measurement 1.3), whereas it was moderate for PHQ-4 item 2 (ICC 0.54 [95% CI 0.40 to 0.66], weighted kappa 0.54 [95% CI 0.38 to 0.70]) (Fig. 3A-D).

Figure 3.
*Continued*

**Figure:**
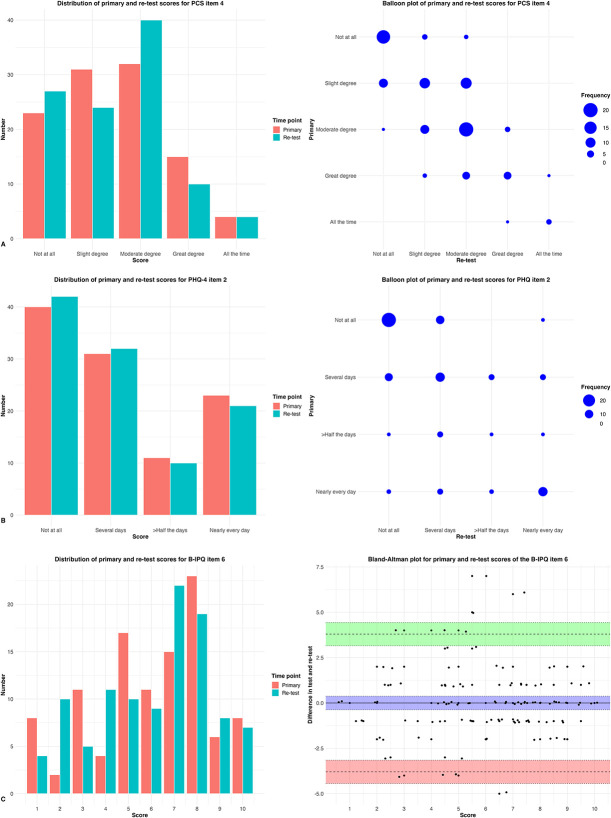

**Figure:**
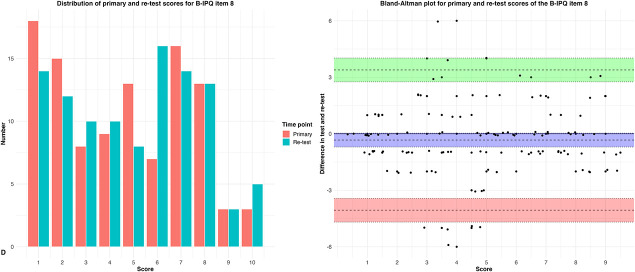

### Response Time

The median total response time of the full PHQ-4, PCS, and B-IPQ was 4 minutes, 6 seconds. When assuming that the response time per item was equal among the questionnaires, the response time per item was 9 seconds for the PHQ-4, 8 seconds for the PCS, and 13 seconds for the B-IPQ. Given these assumptions, our newly developed screening tool has a response time of 43 seconds.

## Discussion

Mental health has gained attention in musculoskeletal healthcare because it influences symptoms, outcomes, and decision-making. Measuring mental health in these patients can be challenging. There is a need for decision support tools that evaluate mental health in musculoskeletal healthcare. We developed a reliable and valid screening tool for pain catastrophizing, psychologic distress, and illness perception that contains only four questions and has an average response time of only 43 seconds. This tool can be used before clinician consultation and may serve as a conversation starter, an expectation management tool, or a decision support tool. For example, it may indicate that further testing is warranted, help guide a clinician when considering referral to a mental health specialist, or support a clinician in choosing between more invasive and less invasive treatments.

### Limitations

A limitation inherent to our observational setting is missing data. However, because our nonresponder analysis suggested there were no clinically relevant differences between responders and nonresponders, we are confident this did not influence our results. Moreover, an advantage of our observational setting is the high ecological validity because our data reflect true daily practice.

Although there are screening tools for specific mental health constructs, there are, to the best of our knowledge, no other screening tools that aim for a combined measure of psychologic distress, pain catastrophizing, and illness perception. A limitation of our method is that we used two measurement instruments that have already been abbreviated (the four-item PHQ-4 for psychologic distress and the B-IPQ for illness perception) to select items for the screening tool. Therefore, our tool should only be used as an indication of one’s mental health, and it should not be considered an in-depth mental health evaluation. However, the high construct validity of our screening tool indicates its items provide a valid view of the constructs of interest. Further, our tool was developed in patients with hand and wrist conditions, and although it seems generalizable, future research might investigate whether the tool can be used in different populations.

The screening tool has no normative values or cutoff scores. Although normative values or cutoff scores can be helpful in clinical decision-making, one may doubt if using these would be appropriate in our screening tool containing only a few questions. The constructs of interest are complex, and the aim of our tool is not to label patients in a certain category. Still, a patient’s scores on our screening tool provide much information that is helpful during clinical consultations, which may provide much context to the patient’s symptoms. Another limitation is that the estimated response time of the screening tool is calculated, not measured, and based on the assumption that the response time per item was equal in the full PCS, PHQ-4, and B-IPQ.

### Discussion of Key Findings

The screening tool could serve as a conversation starter because it may reinforce a clinician’s gut feeling about certain patients and could enable the clinician to discuss the patient’s thoughts and feelings. Hypothetically, this may result in improved patient-reported experiences; for example, a patient may experience more clinician empathy if the clinician is attentive to the patient’s thoughts and feelings. Additionally, it allows the clinician to manage expectations because these thoughts and feelings may affect treatment outcomes. Because of the above, the screening tool may also serve as a decision support tool, because discussing these issues indicates that other treatment choices could be better. For example, if a patient with thumb-base osteoarthritis presents with high pain levels and the mindset screening tool indicates a high degree of pain catastrophizing, a high degree of psychologic distress, and distorted illness perception, this indicates that possibly temporary decreased mental health may explain at least part of the patient’s symptoms. In such case, a purely biomedical approach such as a thumb-base surgery may not be optimal, and less-invasive options may be considered first. Additionally, for example in this case, the screening tool might indicate whether an intervention into mental health may be beneficial, either performed by the clinician or a mental health specialist in more challenging cases. The above will only work if the screening tool is implemented and used, preferably before clinician consultation. Thus, future research could focus on implementing user-friendly data feedback to clinicians, such as through electronic dashboards. Additionally, studies might investigate whether using the tool yields other treatment choices (for example, changes in the ratio of invasive versus noninvasive treatment or the number of referrals to a mental health specialist), differences in outcome expectations, or differences in patient-reported experience measures. In line with this, future studies could also determine whether using the tool leads to better treatment outcomes, such as higher satisfaction with treatment results or increased cost-effectiveness.

The mental health screening tool explained a substantial part of the variance in pain and hand function at baseline and 3 months. This highlights, in line with other studies [3-5, 8, 9, 12, 14-16, 18, 19, 25, 28, 30, 31, 33, 35, 36], the importance of mental health and its relation to treatment decisions and outcomes. The models with only the screening tool items performed nearly as well as the models using the full mental health measures (that is, the entire PCS, PHQ-4, and B-IPQ), which further substantiates the validity of our tool. Using the tool can reduce the time and patient burden of using patient-reported measures yet still collect relevant information for patient care and research.

Our screening tool had high test-retest reliability for most items. Only the PHQ-4 item yielded moderate test-retest reliability. Other studies investigated the test-retest reliability of the PHQ-4 [7, 21] and found better test-retest reliability than we did. However, these studies reported ICCs for the total score of the PHQ-4, whereas we assessed the test-retest reliability specifically for item 2 of the PHQ-4. It seems logical that the test-retest assessment of a single question yields more variability than a total score, because changes at an item level may cancel out at a total score level. Moreover, in our study, the test-retest reliability of this PHQ-4 item may also be affected by the fact that the item specifically asks for the degree of worrying in the past 2 weeks. Thus, hypothetically, the time interval between the test and the retest may also have caused an actual change in that item.

This four-item screening tool has a minimal time burden. If patient-reported measures of mental health are used at all in current daily practice, they are usually only distributed after clinician consultation. Especially with a screening tool that is this short, this is a missed opportunity, because treatment decisions are usually already made in this phase. Our data indicate the screening tool can be reliably used before clinician consultation, which allows the screening tool we developed to be used in daily practice during clinician consultations.

### Conclusion

This ultrashort, valid, and reliable screening tool for mental health (such as psychologic distress, pain catastrophizing, and illness perception) demonstrated added clinical value. The screening tool can be used in daily musculoskeletal healthcare practice as a conversation starter, an expectation management tool, or a decision support tool. For example, the screening tool may indicate that further testing is warranted, guide a clinician in referring to a mental health specialist, or support choices between more invasive and less invasive treatments. Future research could investigate in an experimental setting how this tool can be optimally used and whether using the tool yields other treatment choices or better outcome expectations, patient-reported experience measures, and treatment outcomes.

## Group Authors

Members of the Hand-Wrist Study Group include: RAM Blomme, BJR Sluijter, DJJC van der Avoort, GJ Halbesma, A Kroeze, J Smit, J Debeij, ET Walbeehm, GM van Couwelaar, GM Vermeulen, JP de Schipper, JFM Temming, JH van Uchelen, HL de Boer, KP de Haas, K Harmsen, OT Zöphel, R Feitz, JS Souer, R Koch, SER Hovius, TM Moojen, X Smit, R Hagen, R van Huis, PY Pennehouat, K Schoneveld, YE van Kooij, RM Wouters, J Veltkamp, A Fink, L Esteban Lopez, WA de Ridder, HP Slijper, RW Selles, JT Porsius, J Tsehaie, R Poelstra, MC Jansen, MJW van der Oest, L Hoogendam, JS Teunissen, JE Koopman, J Dekker, MHP ter Stege, JM Zuidam, CA van Nieuwenhoven, CA Hundepool, BEPA van der Heijden, JW Colaris, and WR Bijlsma.

## Supplementary Material
