# Supplementary material for: The Ultrashort Mental Health Screening Tool Is a Valid and Reliable Measure With Added Value to Support Decision-making
Source: Clin Orthop Relat Res. 2023 Jun 20;482(1):59–70. doi: 10.1097/CORR.0000000000002718 (PMC10723896; doi:10.1097/CORR.0000000000002718)
Supplement: Supplementary file 1 [file abjs-482-059-s001.docx]

**Supplemental Table 1.** Nonresponder analysis of Sample 3 (the association with pain and hand function)

| Variable | Responders  (n = 13,061) | Nonresponders  (n = 4,973) | Standardized mean difference |
| --- | --- | --- | --- |
| Age | 55 ± 14 | 51 ± 16 | 0.247 |
| Sex = female | 66 (8602) | 64 (3195) | 0.034 |
| Treatment track  Thumb regular  Thumb extended  Dupuytren  Wrist regular  Wrist extended  Finger regular  Finger extended  Nerve (de-)compression | 15 (1977)  7 (964)  9 (1232)  20 (2669)  8 (1103)  19 (2518)  3 (378)  17 (2220) | 19 (935)  5 (227)  7 (329)  26 (1289)  6 (285)  19 (923)  3 (146)  17 (839) | 0.236 |
| Duration of symptoms in months | 19 ± 39 | 18 (35) | 0.031 |
| Type of work  Unemployed (retired)  Light physical labor (office work)  Moderate physical labor (working in a store)  Heavy physical labor (working in construction) | 35 (4622)  28 (3699)  26 (3371)  11 (1369) | 30 (1488)  29 (1441)  29 (1419)  13 (625) | 0.126 |
| Treated/affected side^a^  Left  Right  Both | 42 (5483)  54 (6995)  5 (583) | 40 (1972)  54 (2666)  7 (335) | 0.103 |
| Dominant hand  Left  Right  Both | 8 (1076)  89 (11,061)  3 (384) | 8 (416)  89 (4438)  2 (119) | 0.034 |
| Second opinion = no | 95 (12,461) | 96 (4769) | 0.024 |
| PHQ-4 total score (scores can range from 0 to 12) | 1.3 ± 2.2 | 1.5 ± 2.4 | 0.073 |
| PCS total score (scores can range from 0 to 52) | 11 ± 9.5 | 11.7 ± 10.2 | 0.065 |
| B-IPQ total score (scores can range from 0 to 80) | 36.8 ± 11.5 | 37.4 ± 11. | 0.047 |

Data are presented as mean ± SD or % (n). Responders are defined as patients who completed the measures of interest at baseline (that is, sociodemographics and mental health questionnaires) and at 3 months of follow-up (the VAS), whereas nonresponders are defined as patients that only completed the measures at baseline. ^a^For the validation set (Sample 2) and the test-retest reliability sample (Sample 4), the patient is asked which side is affected, whereas the values in Samples 1 and 3 reflect the side that is treated.
