# Supplementary material for: The Ultrashort Mental Health Screening Tool Is a Valid and Reliable Measure With Added Value to Support Decision-making
Source: Clin Orthop Relat Res. 2023 Jun 20;482(1):59–70. doi: 10.1097/CORR.0000000000002718 (PMC10723896; doi:10.1097/CORR.0000000000002718)
Supplement: Supplementary file 2 [file abjs-482-059-s002.docx]

**Supplemental Table 2.** Nonresponder analysis of Sample 4 (test-retest reliability)

| Variable | Responders  (n = 105) | Nonresponders  (n = 195) | Standardized mean difference |
| --- | --- | --- | --- |
| Age in years | 56 ± 16 | 55 ± 16 | 0.073 |
| Sex = female | 60 (63) | 61 (119) | 0.021 |
| Duration of symptoms in months | 23 ± 66 | 14 ± 20 | 0.194 |
| Type of work  Unemployed (retired)  Light physical labor (office work)  Moderate physical labor (working in a store)  Heavy physical labor (working in construction) | 42 (44)  19 (20)  29 (30)  11 (11) | 37 (72)  26 (50)  25 (48)  13 (25) | 0.192 |
| Treated or affected side^a^  Left  Right  Both | 33 (35)  37 (39)  30 (31) | 30 (59)  42 (82)  27 (53) | 0.105 |
| Dominant hand  Left  Right  Both | 11 (12)  81 (85)  8 (8) | 10 (19)  82 (159)  9 (17) | 0.065 |
| Second opinion = no | 89 (93) | 89 (173) | 0.019 |

Data are presented as mean ± SD or % (n). Responders are defined as patients who completed the primary test and the retest, whereas nonresponders are defined as patients who only completed the primary test. ^a^For the validation set (Sample 2) and test-retest reliability sample (Sample 4), the patient is asked which side is affected, whereas the values in Samples 1 and 3 reflect the side that is treated.
